# A predictive model combining connectomics and entropy biomarkers to discriminate long‐term vagus nerve stimulation efficacy for pediatric patients with drug‐resistant epilepsy

**DOI:** 10.1111/cns.14751

**Published:** 2024-07-17

**Authors:** Tung‐yang Cheng, Yingbing Hu, Xiaoya Qin, Jiayi Ma, Daqi Zha, Han Xie, Taoyun Ji, Qingzhu Liu, Zhiyan Wang, Hongwei Hao, Ye Wu, Luming Li

**Affiliations:** ^1^ National Engineering Research Center of Neuromodulation, School of Aerospace Engineering Tsinghua University Beijing China; ^2^ Tsinghua‐Berkeley Shenzhen Institute Tsinghua University Shenzhen China; ^3^ Department of Pediatrics Peking University First Hospital Beijing China; ^4^ Pediatric Epilepsy Center Peking University First Hospital Beijing China; ^5^ CAS Key Laboratory of Mental Health, Institute of Psychology Chinese Academy of Sciences Beijing China; ^6^ Department of Psychology University of Chinese Academy of Sciences Beijing China; ^7^ IDG/McGovern Institute for Brain Research at Tsinghua University Beijing China

**Keywords:** drug‐resistant epilepsy, functional connectivity, Hilbert‐Huang spectral entropy, support vector machine, Vagus nerve stimulation

## Abstract

**Aims:**

To predict the vagus nerve stimulation (VNS) efficacy for pediatric drug‐resistant epilepsy (DRE) patients, we aim to identify preimplantation biomarkers through clinical features and electroencephalogram (EEG) signals and thus establish a predictive model from a multi‐modal feature set with high prediction accuracy.

**Methods:**

Sixty‐five pediatric DRE patients implanted with VNS were included and followed up. We explored the topological network and entropy features of preimplantation EEG signals to identify the biomarkers for VNS efficacy. A Support Vector Machine (SVM) integrated these biomarkers to distinguish the efficacy groups.

**Results:**

The proportion of VNS responders was 58.5% (38/65) at the last follow‐up. In the analysis of parieto‐occipital α band activity, higher synchronization level and nodal efficiency were found in responders. The central‐frontal θ band activity showed significantly lower entropy in responders. The prediction model reached an accuracy of 81.5%, a precision of 80.1%, and an AUC (area under the receiver operating characteristic curve) of 0.838.

**Conclusion:**

Our results revealed that, compared to nonresponders, VNS responders had a more efficient α band brain network, especially in the parieto‐occipital region, and less spectral complexity of θ brain activities in the central‐frontal region. We established a predictive model integrating both preimplantation clinical and EEG features and exhibited great potential for discriminating the VNS responders. This study contributed to the understanding of the VNS mechanism and improved the performance of the current predictive model.

## Graphical abstract

The long‐term efficacy of VNS was assessed among 65 pediatric patients. Presurgical EEG analysis shows that VNS responders exhibit higher nodal efficiency in parietal‐occipital EEG α activity and lower entropy in central‐frontal EEG θ activity. The SVM model with clinical and EEG features for VNS efficacy shows high accuracy.

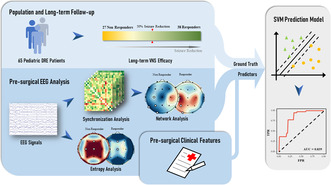

## 1 INTRODUCTION

Vagus Nerve Stimulation (VNS) is an adjuvant therapy for drug‐resistant epilepsy (DRE) and has been proven to be an effective and safe therapy for over 20 years.^1^ The International League against Epilepsy (ILAE) defined DRE as the failure of adequate trials of two tolerated, appropriately chosen, and used antiepileptic drug schedules.^2^ However, the efficacy varies greatly among patients, with only 50%–60% of them being able to achieve more than 50% reduction in seizure frequency, and only about 10%–15% being able to achieve complete control of seizures.^3^ Pediatric patients, in particular, are faced with higher risks during the invasive implantation process, along with higher costs for the surgery and the long‐term course of treatment, which is approximately 1.7 times that of the general population.^4^ Therefore, it will be highly beneficial if preimplantation assessment can identify whether VNS therapy is suitable for a patient or not, thus helping reduce the risk and economic burden.^2^

Previous studies have already explored various biomarkers to predict VNS efficacy. Biomarkers from clinical information included duration of epilepsy, magnetic resonance imaging (MRI) outcome, seizure type, etiology, and so on.^5^, ^6^, ^7^ Other biomarkers included analysis of electroencephalogram (EEG) signals,^8^, ^9^, ^10^, ^11^ cytokine levels,^12^ and tryptophan metabolites.^13^ Also, our previous work revealed heart rate variability (HRV), representing higher vagal cardiac control, to be preimplantation biomarkers of VNS responders.^14^ Specifically, scalp EEG signals, as a critical tool in the diagnosis of epilepsy^15^, ^16^ play an important role in the identification of VNS efficacy biomarkers. Although EEG‐based features such as symmetry,^8^, ^9^ epileptiform discharges,^10^, ^11^ connectomics^17^, ^18^, ^19^, ^20^ and network features^21^ were explored for the preimplantation predictors of VNS efficacy, the results still lack consistency, probably due to the variety of the paradigms of the trials. The effectiveness of all these biomarkers is still uncertain and needs further research. Accordingly, our latest work integrated clinical features and globally averaged EEG synchronization features to establish a predictive model based on Support Vector Machine (SVM) for pediatric VNS responders.^22^ However, it only achieved an accuracy of 75.7% in the 10‐fold cross validation and 61.1% in the external validation cohort.^22^ The limited prediction performance shows the need for a more effective model.

Among all the predictive EEG biomarkers of VNS efficacy, results based on connectomics and network were reported to be the most promising ones,^23^ since epilepsy as a network disease is often characterized by the propagation of hypersynchronous firing of neurons.^24^, ^25^ Studies in this context suggested the possible VNS antiepileptic mechanism as desynchronization and network reorganization of the brain.^26^, ^27^, ^28^ Specifically, functional connectivity (FC) assessments, including phase locking value (PLV), phase lag index (PLI), and weighted PLI (wPLI), were already proven to be related to VNS efficacy.^17^, ^18^, ^19^, ^20^ Network topology analysis further provides a more sophisticated way to look into the relations between brain connectivity and VNS‐induced improvement.^21^

Due to the nonlinear nature of EEG signals, various entropy‐based measures have been employed to quantify their intricate dynamics. Previous studies have observed differences in entropy features across different periods and patterns of seizures.^29^ Notably, abnormal activities or statuses within EEG signals can be readily identified by entropy deviating from the healthy state.^30^ However, the relationship between the efficacy of VNS and entropy remains unclear.

Interestingly, accumulating evidence shows that the effect of VNS on cortical activities seems to be band‐ and region‐specific. Oscillations in the α and θ bands were extensively reported to be significantly affected by chronic VNS, with the influence of α oscillations to be prominent in the central and parieto‐occipital regions,^31^, ^32^, ^33^, ^34^ and θ oscillations in the central‐frontal region.^18^, ^35^ Based on this effect of VNS, we hypothesized that the results of localized analysis in the specific frequency band are potential biomarkers for long‐term VNS efficacy and can improve prediction performance.

In this study, we conducted a follow‐up with an extended duration for identifying VNS responders and nonresponders and further looked into the topological network characteristics based on the EEG synchronization analysis as well as the entropy features. We focused on the pareto‐occipital α activity and central‐frontal θ activity, according to the specific characteristics of the VNS effect mentioned above. Our objective was to identify predictive biomarkers through our analysis, thereby improving the prediction accuracy of our multi‐modal feature‐based predictive model.

## 2 METHODS

### 2.1 Patients recruitment and clinical information acquisition

Eighty‐seven pediatric DRE patients were included in this research. All patients received implantation of VNS (PINS, Beijing, China, or Cyberonics, Houston, TX) between March 2016 and December 2020 in the Pediatric Epilepsy Center of Peking University First Hospital. Inclusion criteria were as follows: (1) aged ≤16 years at seizure onset; (2) diagnosed with DRE; (3) regularly programmed and followed‐up for at least 1 year after VNS implantation; (4) VNS output current ≥1 mA at each follow‐up; (5) preimplantation scalp video‐EEG monitoring recorded for at least 4 h (including awake state ≥40 min) within 6 months prior to implantation.

We excluded 22 patients based on the following exclusion criteria: (1) reception of other antiepileptic therapy after implantation, including surgical resection, ketogenic diet, and so on; use of antiseizure medications (ASMs) not included; (2) termination of VNS therapy; (3) loss of follow‐up; and (4) had irregular/missing seizure frequency recordings. We finally included 65 patients in our research (36 males and 29 females; age at VNS implantation ranging 1.78–15.35 years).

Preimplantation clinical and EEG data were collected retrospectively. Baseline clinical information included (1) demographic information, including gender and body mass index (BMI); (2) age at seizure onset; (3) duration of epilepsy before surgery; (4) seizure type, including generalized, focal, and unknown; (5) seizure frequency; (6) etiology of epilepsy; (7) epilepsy syndrome, including infantile spasms (IS), Lennox‐Gastaut syndrome (LGS), early‐onset epileptic encephalopathy (EOEE), and unclassified syndrome; (8) history of epilepsy resection surgery; (9) number of historical ASMs usage; (10) number of ASMs usage at VNS implantation; and (11) MRI diagnosis. Use of ASMs during the follow‐up period was not restricted, and patients were allowed to add or modify their medications for the most benefit.

Monthly seizure frequency was calculated through telephone follow‐up based on the number of seizures within 3 months prior to the follow‐up time. The efficacy of VNS was quantified and evaluated by calculating the reduction in seizure frequency at the last follow‐up compared to the baseline. Patients who had seizure reduction of ≥50% were considered VNS responders (R50), while others with seizure reduction <50% were labeled as nonresponders (NR50). A VNS efficacy analysis was performed between the two groups. We also calculated the percentage of the patients with over 80% seizure reduction (R80) and with complete seizure relief (R100).

### 2.2 EEG acquisition and preprocessing

Preimplantation scalp video‐EEG signals of the recruited patients were retrospectively acquired, which were recorded within 6 months before implantation, using a 21‐channel EEG system (Neurofax, EEG‐1200C, Japan) positioned according to the 10–20 system placement (Fp1, Fp2, F3, F4, C3, C4, P3, P4, O1, O2, T3, T4, T5, T6, Fz, Cz, Pz, F7, and F8). The data were reviewed by specialists, and events including seizure onset, spikes, and asleep/awake states were marked based on both the electrophysiological and behavioral information.

Awake resting state interictal EEG sections for at least 6 min were identified and selected for each patient. Preprocessing procedures were conducted subsequently with the EEGLAB toolbox (2020) in MATLAB R2018b (Mathworks, Natick, USA). The average reference was implemented during the re‐referencing process, and the sampling rate was set to 500 Hz. A bandpass filtering of 1–30 Hz and a notch filtering of 50 Hz against power‐line interference were applied to the signals. Sections with apparent artifacts or epileptiform discharges were removed by visual inspection. To further eliminate the artifacts, independent component analysis (ICA) was carried out, where components such as eye movement, eye drift, and electrocardiogram (ECG) artifacts were removed.

**FIGURE 1:**
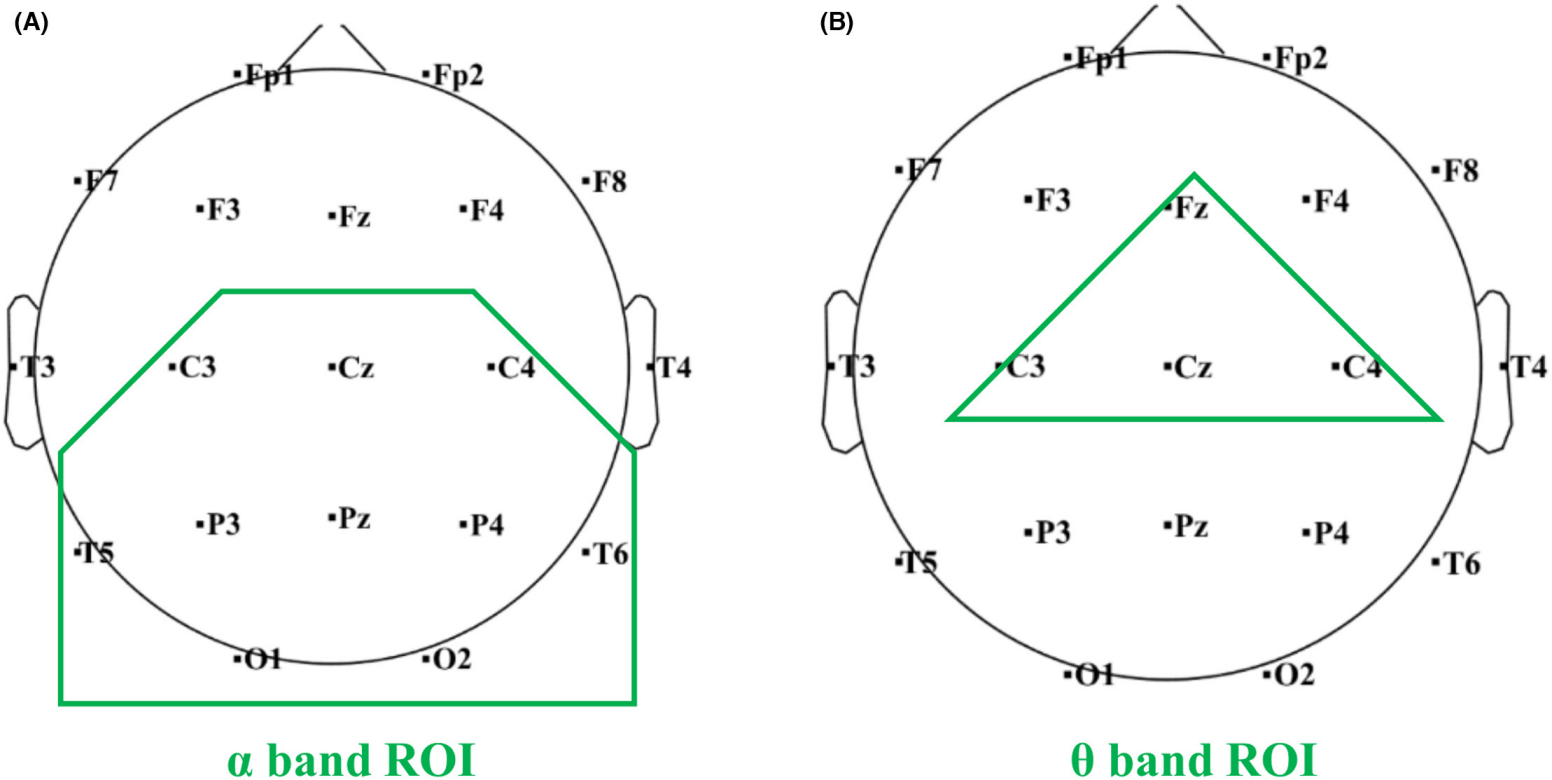
Region of Interest (ROI) in the band‐specific analysis. (A) ROI in the analysis of α band activity. (B) ROI in the analysis of θ band activity.

The preprocessed signals were analyzed in 5 standard EEG frequency bands: δ (1–4 Hz), θ (4–8 Hz), α (8–13 Hz), low β (13–20 Hz), and high β (20–29 Hz). According to the band‐ and region‐specific effect of VNS influence in the EEG signals, a region of interest (ROI) was defined separately in the analysis of α and θ activity. ROI of the α activity analysis included C3, C4, Cz, T5, T6, P3, P4, Pz, O1, and O2, located in the parieto‐occipital region (Figure 1A). ROI of the θ activity analysis included Fz, Cz, C3, and C4 located in the central‐frontal region (Figure 1B).

### 2.3 EEG feature extraction

#### 2.3.1 EEG FC measures and network topological analysis

The EEG phased‐based FC analysis of PLI was included in our study to quantify the phase synchronizations. It was calculated based on the phase difference,^36^ defined as

$$\mathrm{PLI}=\left|\frac{1}{N}\sum_{n=1}^{N}\mathrm{sign}\left(∆\phi_{t_{n}}\right)\right|$$

where N represents the number of time points and $∆\phi_{t_{n}}$ represents the phase difference between the two signals at $t_{n}$, based on the Hilbert transform of the full‐length signals. The PLI was calculated in different frequency bands in a 2‐s length unit for averaging between every two channels of the EEG signals, and was demonstrated in the form of an FC matrix. To exclude the possible influence of the heterogeneity of the signal, we randomly selected 20% of the total number of PLI values 5 times with replacement after the calculation of the indicators. The final results of the synchronization indicators were presented as the mean of the values of the selected epochs.

All topological network analysis was conducted using the GRETNA package (http://www.nitrc.org/projects/gretna/). The PLI matrix calculated above worked as the adjacency matrix, which was considered a weighted network with a subject‐specific threshold.^37^ The threshold was set to be sparsity based, where the number of the edges divided by the maximum possible number of the edges in the network was set to be a fixed ratio, and values below the threshold were set to be 0, as shown below:

$$W_{\mathrm{ij}}=\left[w_{\mathrm{ij}}\right]=\left\{\begin{array}{c} \left|c_{\mathrm{ij}}\right|,\text{if}\left|c_{\mathrm{ij}}\right|>r_{\text{threshold}}\\ 0,\text{others} \end{array}\right.$$

where $C_{\mathrm{ij}}=\left[c_{\mathrm{ij}}\right]$ is the original adjacency matrix, and $r_{\text{threshold}}$ is the subject‐specific threshold of the matrix.

Network features were calculated based on the distance between two nodes. Considering the possible infinite distance value for some disconnected nodes, the efficiency between nodes is applied to attain a finite value. The average of the efficiency between any two nodes is the Global Efficiency,^37^ calculated as

$$\mathrm{GE}=\frac{1}{\frac{1}{2}N\left(N-1\right)}\sum_{i\geq j}\frac{1}{d_{\mathrm{ij}}}$$

where $d_{\mathrm{ij}}$ is the distance value between two nodes.

Nodal efficiency was also calculated for each specific node to quantify its communication with the whole network.^38^

#### 2.3.2 EEG entropy analysis

Hilbert‐Huang Spectral Entropy (HHSE) is a time‐frequency entropy analysis based on the Hilbert‐Huang Transform (HHT), which uses the empirical mode decomposition (EMD) method to deal with nonlinear and nonstationary signals.^39^ For the EEG signal $x\left(t\right)$, it can be decomposed by EMD into a set of intrinsic mode functions (IMFs) and the residue $r_{n}\left(t\right)$ as:

$$x\left(t\right)=\sum_{i=1}^{n}\mathrm{IMF}{\left(t\right)}_{i}+r_{n}\left(t\right)$$

Then the analytic signal of each IMF is obtained through Hilbert transformation as:

$$Z_{i}\left(t\right)={\mathrm{IMF}}_{i}\left(t\right)+\mathrm{jH}\left[{\mathrm{IMF}}_{i}\left(t\right)\right]=a_{i}\left(t\right)e^{j\int \omega_{i}\left(t\right)dt}$$

where $a_{i}\left(t\right)$ and $\omega_{i}\left(t\right)$ represent the instantaneous amplitude and frequency.

The Hilbert‐Huang amplitude spectrum can then be constructed by all the IMFs as a function of frequency ω and time t:

Hωt=Re∑i=1Naitej∫ωitdt

The marginal spectrum of the frequency $h\left(f\right)$ is constructed by the integration of $H\left(f,t\right)$ over time t, and is normalized into $\hat{h}\left(f_{0}\right)$ for each frequency $f_{0}$, as shown below:

$$h\left(f\right)=\int H\left(f,t\right)$$

$$\hat{h}\left(f_{0}\right)=\frac{h\left(f_{0}\right)}{\sum_{f}h\left(f\right)}$$

To further investigate the entropy characteristics of specific EEG activity in a certain frequency band, we implemented the local HHSE.^40^ For the interested frequency band F, the normalized local marginal spectrum is defined as:

$${\hat{h}}_{\mathrm{loc}}\left(f_{0}\right)=\frac{h\left(f_{0}\right)}{\sum_{f\in F}h\left(f\right)}$$

Thus, the local HHSE can be calculated by the definition of Shannon entropy algorithm:

$${\text{HHSE}}_{\mathrm{loc}}=-\sum_{f\in F}{\hat{h}}_{\mathrm{loc}}\left(f\right)\ln{\hat{h}}_{\mathrm{loc}}\left(f\right)$$

### 2.4 Machine learning for the prediction

#### 2.4.1 Feature selection process

All the features were normalized between 0 and 1 through min‐max normalization across the study population to avoid the bias resulting from the huge difference in scales.

Feature selection was applied to eliminate redundant or irrelevant features. We applied a wrapper method of recursive feature elimination with cross validation (RFECV), which assessed the features iteratively based on the performance of the learning method.^41^ The feature selection algorithm sifted out the optimal feature combination under a specified number of features, and the optimal number of features with the highest cross‐validation score was determined.

#### 2.4.2 Learning model for efficacy prediction

We used the linear Support Vector Machine (SVM) to conduct the binary classification between the R50 and NR50. The model was trained and validated through nested cross validation (nested‐CV), which contained an inner loop and an outer loop. The inner loop applied a grid‐search strategy to the model parameters to reach the optimum, while the fold in the outer loop served as the validation cohort independent from the discovery cohort. A 5‐fold inner loop and a 10‐fold outer loop were used in this article.

The performance of the model was assessed based on the mean result of the nested‐CV. The confusion matrix and receiver operating characteristic (ROC) curve were applied, and accuracy, precision, specificity, and area under the ROC curve (AUC) were calculated. A permutation test based on comparisons to classifications with random labels was performed.

### 2.5 Statistical analysis

Clinical features between the R50 and NR50 were analyzed using the Mann‐Whitney *U* test for continuous variables and chi‐square analysis or Fisher's exact test for nominal variables. Electrophysiological features were analyzed between two efficacy groups with the Mann‐Whitney *U* test in each frequency band, and Cohen's *d* was calculated to quantify the size effect. The *p*‐values of all the nodal results were corrected with a false discovery rate (FDR). The data were shown as the mean ± standard deviation (SD). Statistical analysis was performed with MATLAB 2018b. A significance level of 0.05 was set.

## 3 RESULTS

### 3.1 Clinical outcomes

In our cohort of 65 pediatric DRE patients, the median follow‐up time was 3.51 years (range 1.78–5.95 years). The proportions of R50 group, R80 group, and R100 group were 58.5% (38/65), 47.7% (31/65), and 13.8% (9/65), respectively.

The results of the baseline clinical features between the R50 and NR50 groups are presented in Table 1. No significant difference was found in clinical features between the R50 and NR50 groups at baseline.

**TABLE 1:** Baseline clinical features between the R50 and NR50 groups.

Baseline (*n* = 65)	NR50 (*n* = 27, 41.5%)	R50 (*n* = 38, 58.5%)	*p‐*value
Seizure frequency, times/month	537.80 ± 779.21 (20–3000)	875.31 ± 1280.99 (4–5430)	0.558
BMI, kg/m^2^	17.63 ± 4.04 (12.45–29.33)	16.34 ± 2.70 (11.76–23.14)	0.247
Age of seizure onset, years	2.22 ± 2.42 (0–8.65)	2.47 ± 2.98 (0–11.68)	1.000
Duration of epilepsy before implantation, years	3.67 ± 2.57 (0.66–10.03)	3.37 ± 1.61 (0.94–9.22)	0.852
Age at VNS implantation, years	5.90 ± 3.71 (1.78–15.35)	5.84 ± 3.20 (1.91–14.99)	0.637
Number of ASMs at baseline, *n*	2.93 ± 1.24 (0–5)	2.95 ± 0.98 (0–4)	1.000
Number of historical ASMs, *n*	5.74 ± 2.09 (2–9)	6.13 ± 1.91 (3–10)	0.701
History of previous epilepsy surgery	3 (11.11%)	6 (15.79%)	0.862
Gender			
Male	16 (59.26%)	20 (52.63%)	0.782
Female	11 (40.74%)	18 (47.37%)	
Etiology of epilepsy			
Structural	16 (59.26%)	18 (47.37%)	0.781
Immune	1 (3.70%)	1 (2.63%)	
Genetic	1 (3.70%)	2 (5.26%)	
Unknown	9 (33.33%)	17 (44.74%)	
Predominant seizure type			
Generalized	12 (44.44%)	15 (39.47%)	0.884
Focal	18 (66.67%)	23 (60.53%)	0.807
Unknown	15 (55.56%)	24 (63.16%)	0.719
Epilepsy syndrome			
Infantile Spasm	4 (14.81%)	10 (26.32%)	0.754
Lennox–Gastaut syndrome	2 (7.41%)	3 (7.89%)	
EOEE	3 (11.11%)	5 (13.16%)	
Unclassified	18 (66.67%)	22 (57.89%)	
MRI			
Multifocal	13 (48.15%)	15 (39.47%)	0.651
Focal	5 (18.52%)	6 (15.79%)	
Negative	9 (33.33%)	17 (44.74%)	

*Note*: Continuous variables are shown in mean ± SD (range), and nominal variables are shown in *n* (%).

### 3.2 EEG α band activity outcomes in ROI

**FIGURE 2:**
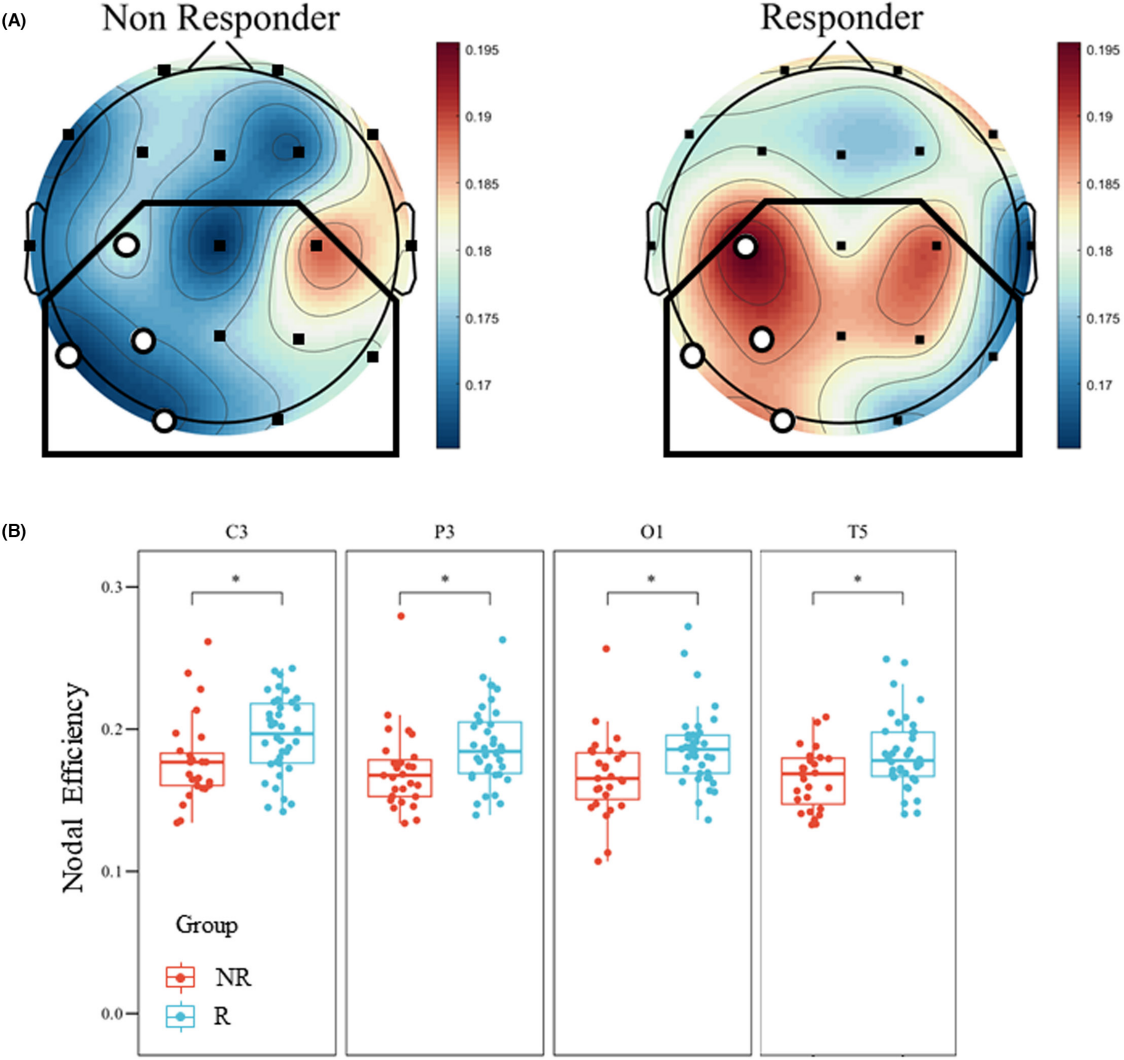
Results of the α band analysis. (A) Mapping of the nodal efficiency based on the α band PLI matrix. The region of interest was marked with black frame. Electrodes with a significant difference were marked with hollow dots. Results demonstrated significantly lower nodal efficiency in NR50 than R50 in 4 electrodes (C3, T5, P3, and O1) within the ROI, especially located in the left hemisphere. (B) Box plot of nodal efficiency in channels with a significant difference between NR50 and R50 in α band within the ROI.

The global topological results in the α band showed a significant difference between the two groups in global efficiency (Figure 2A, NR50: 0.175 ± 0.010 vs. R50: 0.181 ± 0.014, *p* = 0.047). As for the nodal features, results demonstrated significantly lower nodal efficiency in the NR50 group than in the R50 group in 4 electrodes (C3, T5, P3, and O1) within the ROI (Figure 2B, Table 2), primarily concentrated in the left hemisphere. The results in connectomics (Figure S1) and network analysis showed a higher ROI synchronization state and a stronger ability to convey information about α activity for responders.

**TABLE 2:** Baseline electrophysiological characteristics between the R50 and NR50.

Electrophysiological Characteristics	NR50 (*n* = 27, 41.5%)	R50 (*n* = 38, 58.5%)	*p‐*value	Cohen's *d*
*α band features*				
Global Efficiency	0.175 ± 0.010	0.181 ± 0.014	0.047	−0.496
Nodal Efficiency of C3	0.178 ± 0.030	0.195 ± 0.028	0.033	−0.624
Nodal Efficiency of C4	0.188 ± 0.026	0.189 ± 0.029	0.785	−0.033
Nodal Efficiency of P3	0.171 ± 0.029	0.188 ± 0.028	0.025	−0.605
Nodal Efficiency of P4	0.180 ± 0.023	0.185 ± 0.027	0.715	−0.216
Nodal Efficiency of O1	0.167 ± 0.029	0.186 ± 0.027	0.025	−0.696
Nodal Efficiency of O2	0.175 ± 0.022	0.174 ± 0.037	0.761	0.055
Nodal Efficiency of T5	0.165 ± 0.021	0.183 ± 0.026	0.038	−0.726
Nodal Efficiency of T6	0.178 ± 0.020	0.173 ± 0.029	0.242	0.216
Nodal Efficiency of Cz	0.165 ± 0.024	0.181 ± 0.032	0.070	−0.557
Nodal Efficiency of Pz	0.175 ± 0.028	0.185 ± 0.032	0.497	−0.318
Mean HHSE of ROI	2.367 ± 0.038	2.375 ± 0.043	0.284	0.506
*θ band features*				
Global Mean HHSE	1.729 ± 0.024	1.709 ± 0.049	0.061	0.495
Mean HHSE of ROI	1.732 ± 0.024	1.713 ± 0.046	0.031	0.517

*Note*: Results are presented as mean ± standard deviation.

Results in the analysis of α band HHSE did not show a significant difference in the global mean value (Figure S2, NR50: 2.3681 ± 0.0389 vs. R50: 2.3783 ± 0.0449, *p* = 0.124). Results in α band within the ROI also showed no significant difference between the two groups (Table 2).

### 3.3 EEG θ band activity outcomes in ROI

The PLI matrix of the EEG θ band activity did not exhibit a general group difference, with only 3 connections showing minor levels of significance (Figure S1). Based on this result, no further investigation was pursued in terms of network topological analysis.

**FIGURE 3:**
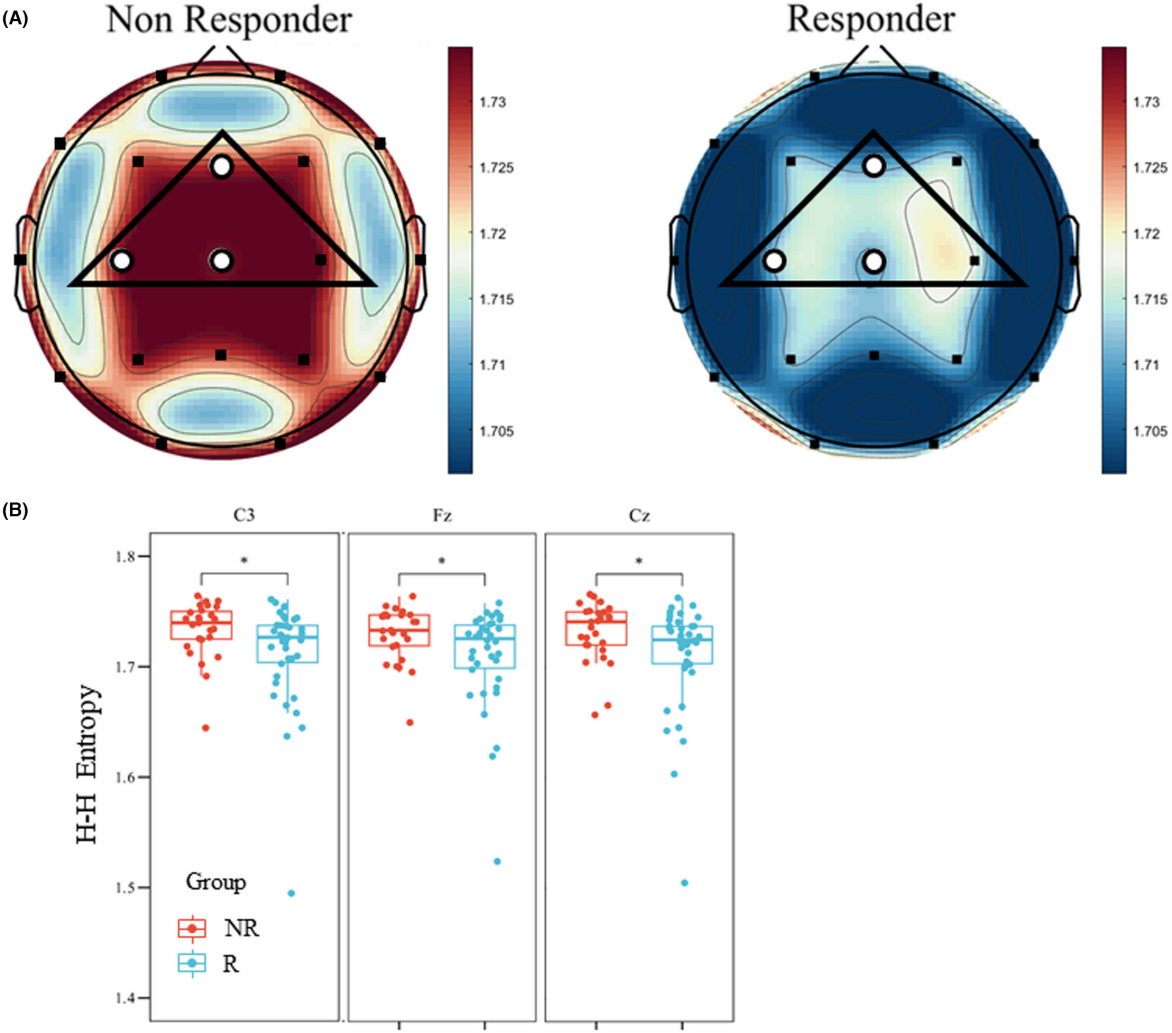
Results of the θ band analysis. (A) Mapping of the HHSE in the θ band. The region of interest was marked with black frame. Electrodes with a significant difference were marked with hollow dots. Results demonstrated significantly higher nodal efficiency in the NR50 group than in the R50 group in 3 electrodes (C3, Fz, and Cz) within the ROI. The results indicated a more significant difference in the central region. (B) Box plot of θ band HHSE in channels with significant difference between NR50 and R50 in θ band within the ROI.

Results in the analysis of θ band HHSE did not show a significant difference in the global mean value (Figure 3A, NR50: 1.729 ± 0.024 vs. R50: 1.709 ± 0.049, *p* = 0.061). However, nodal results of the θ band HHSE indicated a significantly higher value in the NR50 group than the R50 group in 3 electrodes (Fz, C3, and Cz) within the ROI (Figure 3B, Table 2). The mean result of the θ band HHSE within ROI showed a significant difference between the NR50 group and the R50 group (Table 2, NR50: 1.732 ± 0.024 vs. R50: 1.713 ± 0.046, *p* = 0.031). The results of the HHSE demonstrated the preimplantation entropy state of θ activity in responders with a higher regularity and lower complexity within the ROI.

### 3.4 Prediction model based on the clinical information and EEG signals

Thirty‐one features analyzed in the previous sections were included in the model: (1) Twenty clinical features, with categorical variables transformed into dummy variables through one‐hot coding; (2) Ten topological network features, including the nodal efficiency of all the ROI electrodes based on α band PLI; (3) 1 HHSE feature, which was the mean entropy value of the ROI in θ band. The correlation matrix of these features revealed that there were no absolute correlation values greater than 0.8 between any two features (Figure S3), which indicated that these features captured a diverse range of information.

**FIGURE 4:**
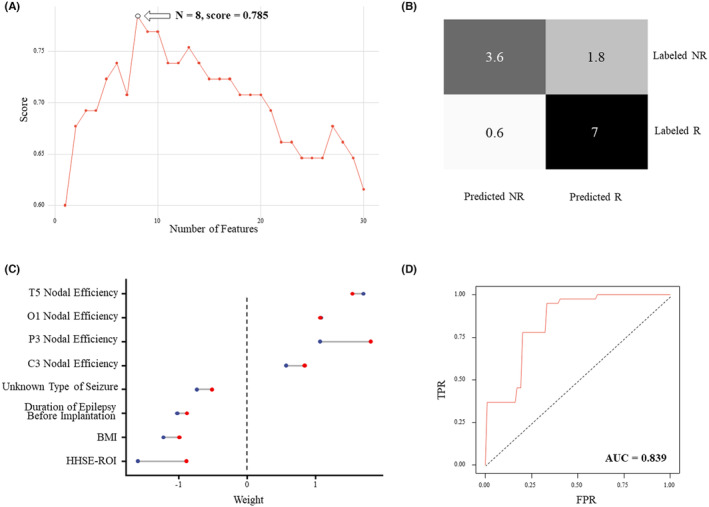
Results of the prediction model. (A) Cross‐validation Scores with different feature numbers during the feature selection process. The combination of 8 features (duration of epilepsy, BMI, unknown type of seizure; nodal features in C3, T5, P3, O1; HHSE feature) was sifted out to have the highest score of 0.785. (B) Confusion Matrix of the Nested Cross Validation of Support Vector Machine. (C) Range of the features' weights from all folds during cross validation. Clinical and HHSE features showed negative values of weights, while the nodal efficiency features exhibited positive values of weights. (D) Receiver Operating Characteristic (ROC) of the model. The area under the curve (AUC) was 0.839.

The feature selection process sifted out 8 features (clinical features of duration of epilepsy, BMI, unknown type of seizure; nodal features in C3, T5, P3, O1; HHSE feature), the combination of which had an highest score (Figure 4A). All the EEG features selected had the absolute Cohen's *d* value over 0.5 (Table 2). The linear SVM model generated from these features through nested‐CV achieved an outcome with an accuracy of 81.5% and a precision of 80.1%%. The confusion matrix and ROC of the model are relatively exhibited in Figure 4B,D, and the AUC value of the ROC was calculated to be 0.839. The result of the permutation test supported the effectiveness of the model's prediction power (*p* < 0.001).

The weights of the features in the final model from the 5 outer folds are shown as a range from min to max (Figure 4C). The topological network features all had positive weights, indicating that the R50 group has a higher tendency to have more synchronized α band EEG activity in the electrodes selected, while the clinical features and HHSE feature all had negative weights, indicating potential adverse effects on the prognosis of VNS from these factors.

## 4 DISCUSSION

This study observed the long‐term improvement of pediatric VNS efficacy and pinpointed the biomarkers from clinical information and EEG signals to establish a predictive model. It is noteworthy that the duration of the follow‐up time affected the results significantly. For the 65 patients included in this study, compared to our prior research,^22^ the median follow‐up time extended from 2.31 years (range 1.04–4.68 years) to 3.51 years (range 1.78–5.95 years). The proportion of the R50 group increased from 53.8% (35/65) to 58.5% (38/65); the R80 group increased from 43.1% (28/65) to 47.7% (31/65); and the R100 group remained at 13.8% (9/65). Along with this improvement in therapeutic outcome, our major conclusion in EEG analysis also changed. Instead of the significant results of synchronization in the high β band from our prior work,^22^ we only found a similar characteristic in the α band in this work. We believed that a follow‐up time of at least 2 years enabled a relatively comprehensive observation of the long‐term VNS efficacy. In 1999, Morris et al. followed up with 440 patients and found that the proportions of responders at 1 year, 2 years, and 3 years after implantation were 36.8%, 43.2%, and 42.7%, respectively, and the median reduction rates in seizure frequency were 35%, 44.3%, and 44.1%, respectively,^42^ indicating that efficacy gradually improved within the first 2 years after implantation and remained stable thereafter. A long‐term observation of VNS efficacy found that 89.5% of the responders experienced a positive response within a two‐year timeframe, with continued and steady improvement over the long term.^43^ In another research, the response rates among pediatric patients were reported to be 55%, 60%, and 52% at 1 year, 2 years, and 4 years of follow‐up, respectively.^44^ The uncertainty and instability of the VNS efficacy lead to the necessity of a more comprehensive follow‐up procedure to assess the long‐term responsiveness. In our prior research,^22^ only 55.7% of the patients received a follow‐up longer than 2 years. Based on a long‐term follow‐up, our results provided evidence for early identification of DRE children with long‐term response to VNS, with 86.4% of the patients having a follow‐up time over 2 years, which makes sure that the response observed is more reasonable.

In our study, the R50 group's higher nodal efficiency and greater connectivity of parieto‐occipital α activity as well as lower entropy of central‐frontal θ activity suggested potential baseline predictive biomarkers for pediatric VNS efficacy. This frequency‐ and region‐specific characteristic of the VNS effect has been reported in previous studies. For α activity, the attenuating effect of VNS on this brain activity was frequently reported.^31^, ^32^, ^33^, ^34^ Kavakbasi et al.^32^ found a reduction in the parieto‐occipital MEG α band power during acute VNS. A recent study demonstrated that transcutaneous VNS (tVNS) can induce attenuation in occipital EEG α‐band oscillation.^34^ As for θ oscillations, VNS can also have a significant influence on certain regions.^18^, ^21^, ^35^ Vespa et al.^18^ found that VNS responders exhibited stronger sleep EEG desynchronization of θ band activity in the central and left frontal regions. Marrosu et al.^35^ reported a significant reduction in inter‐ and intrahemispheric coherence of the θ band in the central region under VNS. In short, VNS tends to affect specific frequency bands in certain regions of brain activity.

The network analysis and connectivity estimation of parieto‐occipital α oscillations in the two groups indicated VNS's major effect on epilepsy. Topological network results showed higher nodal efficiency in the R50 group than in the NR50 group, which aligned with VNS's network reorganization effect. Connectivity estimation showed higher synchronization in the R50 group, which revealed the VNS's desynchronization effect of brain activity. These two effects have been suggested to be the major mechanism of VNS^26^, ^27^, ^28^ and have been discussed in the search for predictive biomarkers.^17^, ^18^, ^19^, ^20^, ^21^ Vespa et al.^18^ found that network efficiency was significantly decreased in sleep by the acute VNS effect in responders, which exhibited VNS's modulation of epileptogenic networks. Furthermore, Bodin et al.^17^ found a lower postsurgical α band synchronization level in VNS responders than nonresponders, which proved the frequency‐specific characteristic of the VNS desynchronization effect. Based on our results, we inferred that the high baseline network efficiency and synchronization level of the responders might render these patients more susceptible to being influenced by the network reorganization and desynchronization effect of VNS more profoundly, thus leading to a more pronounced therapeutic response than the nonresponders.

The findings in the θ band entropy analysis also implied VNS's desynchronization effect on brain activity. Entropy measures can interpret information capacity, especially when the brain system approaches critical‐state dynamics.^45^, ^46^ In the analysis of time‐frequency‐based entropy analysis in epilepsy, previous studies have already proved that ictal signals present a most regular pattern, characterized by lower entropy values compared to the healthy and interictal states.^30^, ^46^, ^47^ This highly ordered feature of epileptic EEG signals during seizures suggested its relation with the synchronization characteristic of this period. Previous research has already proved the relationship between brain FC and entropy, with regional entropy negatively correlated with FC level.^48^ Our results of FC and entropy analysis are consistent in this context, with responders exhibiting higher synchrony and lower entropy, again suggesting greater potential improvement through VNS's modulation than nonresponders.

The different characteristics of the VNS efficacy groups in the region‐specific α and θ activities could reflect their potential relationship and thus help clarify the VNS mechanism. The stimulation of the vagus nerve may influence these activities through the activation of the locus coeruleus‐noradrenaline (LC‐NE) system, which results in modulation of perception‐driven behaviors and suppression of spontaneous activities across multiple sensory cortices.^49^ Pupil dilation with α attenuation after VNS was also explained to be mediated by this mechanism with increased arousal activation.^34^ Besides, as indexes of comprehensive sensory‐cognitive function, α and θ activities seem to react to stimulation conjointly. Upon visual stimulation, while α activity is enhanced the most at central, parietal, and occipital sites,^50^ highly ordered and phase‐locked θ component dominance in central‐frontal locations also occurred subsequently during the greatest reduction of wavelet entropy.^51^ Our findings of the difference between the two groups may illustrate how activation of the LC‐NE system by VNS reshapes the cortices across multiple regions of the brain and how the reorganization of the system improves the treatment of the DRE.

With the long‐term follow‐up and the integration of a more detailed analysis of the EEG signal, our model successfully predicted the long‐term pediatric VNS efficacy with improved performance. Previous prediction models based on machine learning algorithms are still rare. The research with the best performance developed a multimodal connectomic prediction algorithm for pediatric DRE patients using diffusion tensor imaging (DTI) and DTI‐informed MEG, demonstrating an accuracy of 89.5%.^52^ However, this assessment is highly costly or unavailable for most institutions. Our model, on the other hand, with the integration of clinical and EEG data, not only demonstrates promising validation results, but is also accessible in clinical practice for most institutions. We reached an accuracy of 81.5%, a precision of 80.1%, and an AUC of 0.838, which is an improvement of our previous work.^22^ This precision means that 80.1% of the patients sifted by this model are expected to respond to the therapy, which is higher than the overall responder rate. The weights of the selected features indicated a possible correlation between the preimplantation information and long‐term VNS efficacy. Specifically, pediatric DRE patients with a longer duration of epilepsy before implantation, a higher BMI, or suffer from an unknown seizure type as a major seizure type tend to have a bad prognosis for VNS implantation. This information can not only help us identify the potential candidate for VNS implantation, but also help us understand why patients respond differently to the therapy. Further optimization of the model should draw us closer to the eventual clinical practice for an accurate VNS presurgical assessment.

Our study has several limitations. First, our model requires an independent validation cohort consisting of multicenter samples. Though nested cross‐validation (nest‐CV) realizes validation within the discovery cohort, it cannot provide sufficient evidence for the generalizability of the model. Second, the size of the dataset is relatively small and may introduce bias. Future studies should aim to collect a larger multicenter dataset to more comprehensively represent population heterogeneity and enhance the model's performance. Then, the spectral differences found in this research are also affected by age and the localization of seizure foci. This may influence the interpretation of the spectral differences between the efficacy groups. Finally, our study solely employed a linear SVM model. Exploring the application of additional classification algorithms in this context may potentially yield better performance.

## AUTHOR CONTRIBUTIONS

Tung‐yang Cheng: Conceptualization; methodology; investigation; formal analysis, writing ‐ original draft. Yingbing Hu: Conceptualization; methodology; formal analysis; writing ‐ original draft. Xiaoya Qin: Formal analysis. Jiayi Ma: Investigation; formal analysis. Daqi Zha: Formal analysis. Han Xie: Investigation. Taoyun Ji: Investigation. Qingzhu Liu: Investigation. Zhiyan Wang: Conceptualization; methodology; formal analysis; review & editing. Hongwei Hao: Review & editing; supervision. Ye Wu: Review & editing; supervision. Luming Li: Review & editing; supervision.

## CONFLICT OF INTEREST STATEMENT

None of the authors has any conflict of interest to disclose.

## Supporting information


Figure S1.

## Data Availability

The data that support the findings of this study are available on request from the corresponding author. The data are not publicly available due to privacy or ethical restrictions.
