# Supplementary material for: A predictive model combining connectomics and entropy biomarkers to discriminate long‐term vagus nerve stimulation efficacy for pediatric patients with drug‐resistant epilepsy
Source: CNS Neurosci Ther. 2024 Jul 17;30(7):e14751. doi: 10.1111/cns.14751 (PMC11252558; doi:10.1111/cns.14751)
Supplement: Supplementary file 1 — Figure S1. [file CNS-30-e14751-s001.docx]

# Supplemental Figures


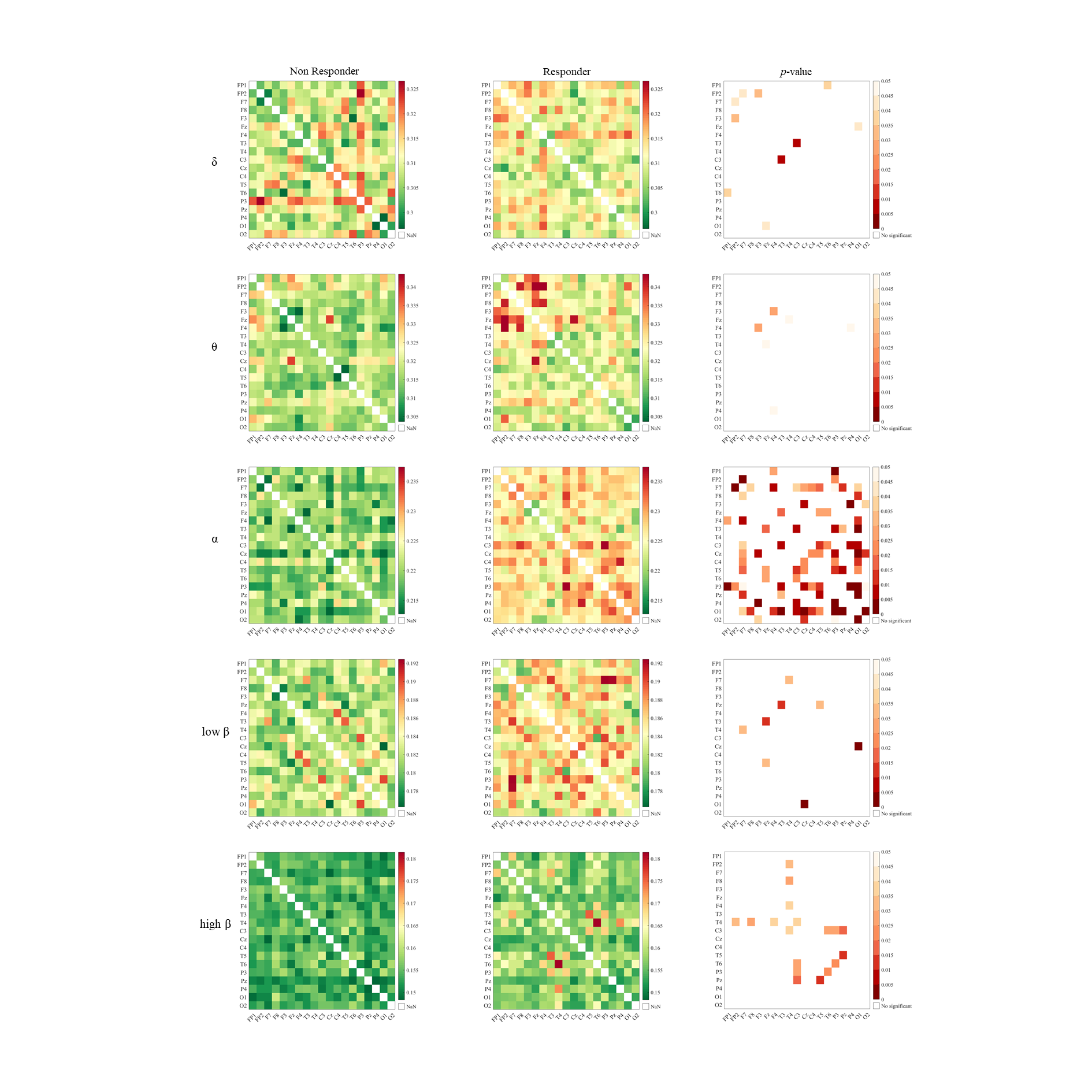


**Figure S1**

PLI matrix averaged over non-responders (left column) or responders (middle column) in δ, θ, α, low β and high β band. The right column shows significant *p*-values (*p*<0.05) of Mann–Whitney U test between responder group and non-responder group in PLI matrix with all the frequency bands. Results showed that only the PLI matrix in α band demonstrated a general significant difference between NR50 and R50 among the connections between electrodes.


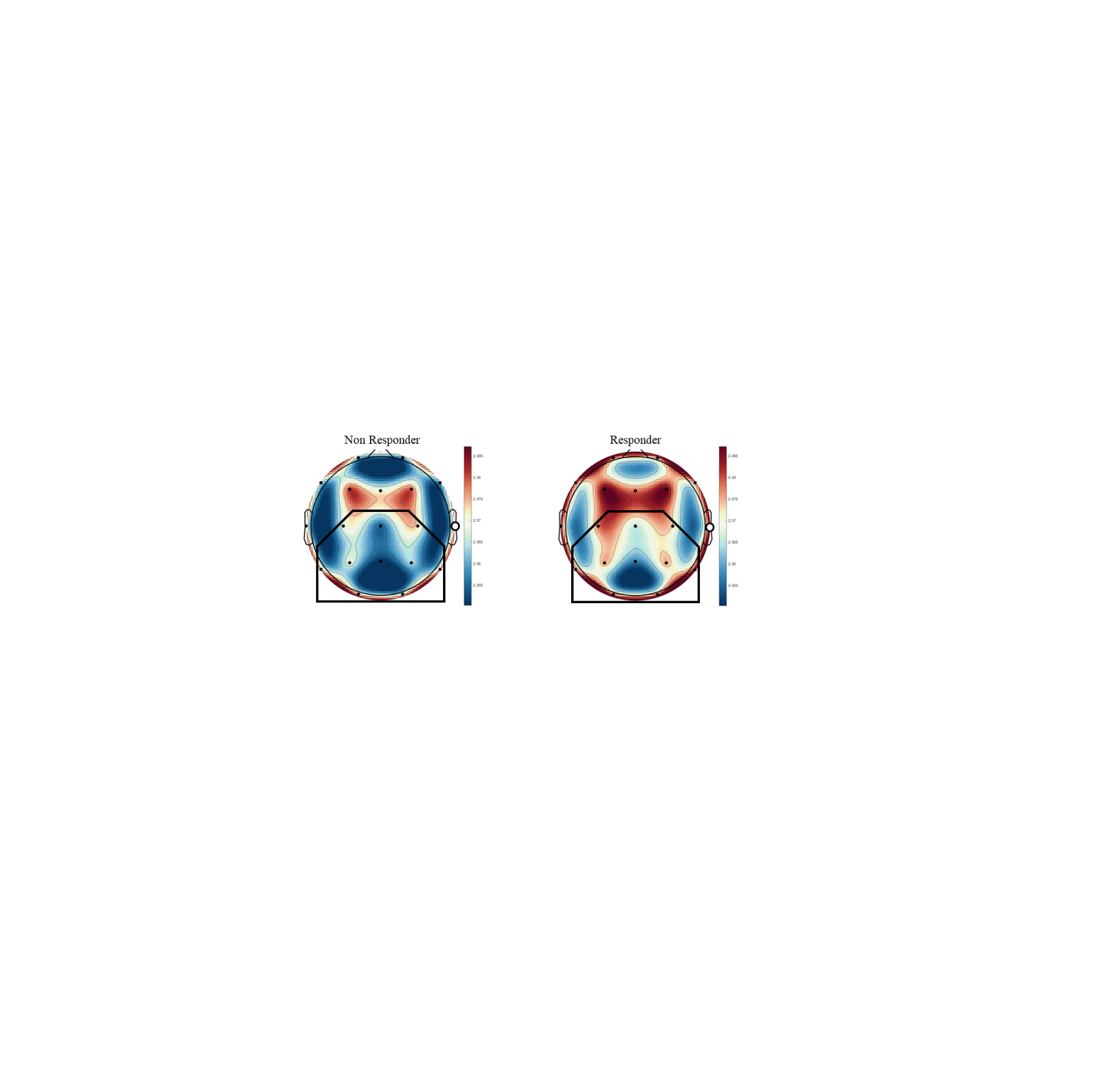


**Figure S2**

Mapping of the HHSE in α band. Region of Interest was marked with black frame. No electrode in the ROI exhibits significant difference between NR50 and R50.


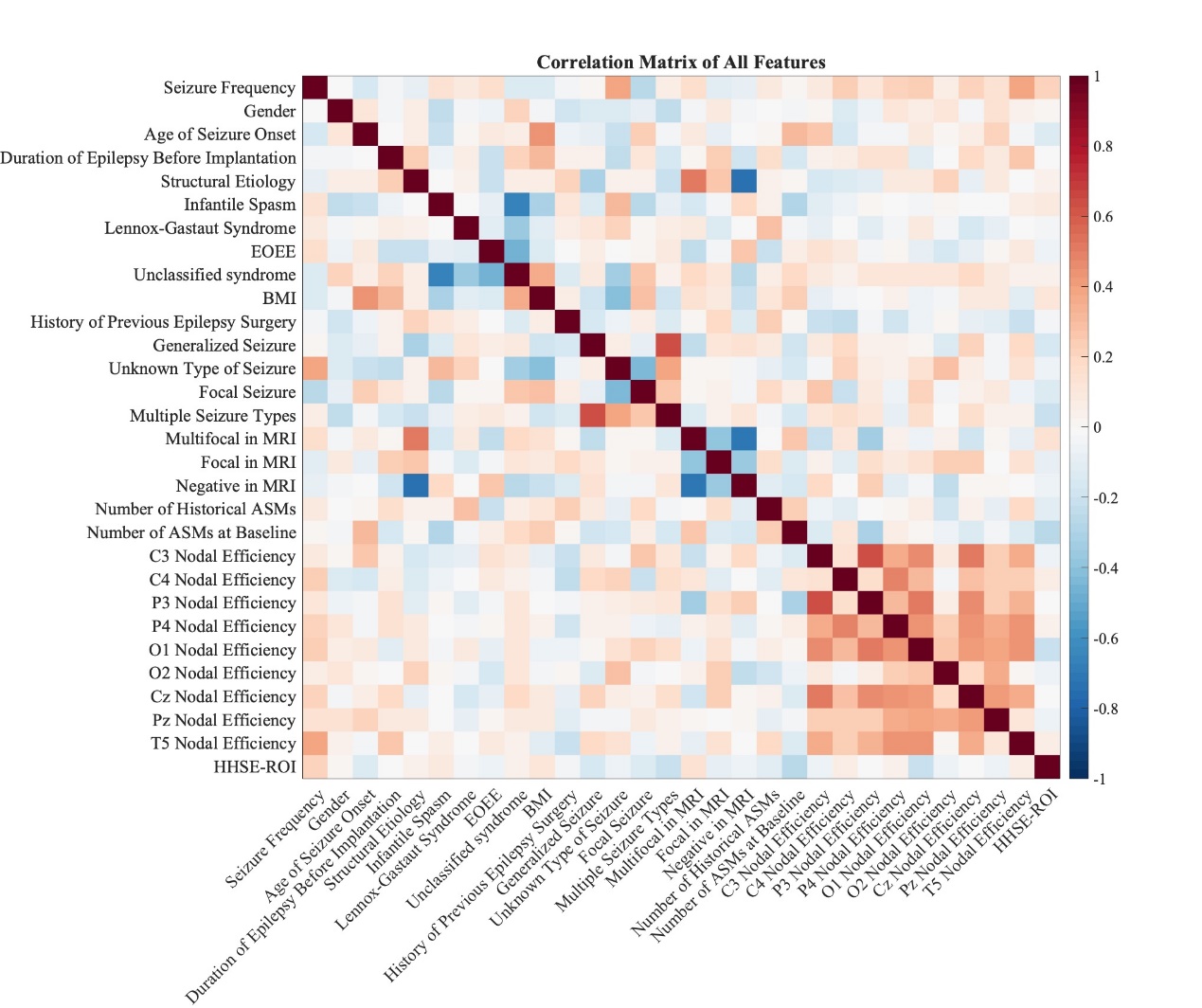


**Figure S3**

Correlation matrix of all features included in the model. No absolute value of the correlation efficient over 0.8 were found between any two features.
